# Highly porous nanoberyllium for X-ray beam speckle suppression

**DOI:** 10.1107/S1600577515003628

**Published:** 2015-04-09

**Authors:** Alexander Goikhman, Ivan Lyatun, Petr Ershov, Irina Snigireva, Pawel Wojda, Vladimir Gorlevsky, Alexander Semenov, Maksim Sheverdyaev, Viktor Koletskiy, Anatoly Snigirev

**Affiliations:** a Immanuel Kant Baltic Federal University, Nevskogo str. 14, Kaliningrad 236041, Russian Federation; b European Synchrotron Radiation Facility, BP 220, 38043 Grenoble, France; c Gdańsk University of Technology, 11/12 G. Narutowicza, Gdańsk 80-233, Poland; d A. A. Bochvar High-Technology Scientific Research Institute for Inorganic Materials, Rogova str. 5a, Moscow 123098, Russian Federation

**Keywords:** porous nanoberyllium, X-ray speckle, X-ray imaging, X-ray microscopy

## Abstract

This paper reports a special device called a ‘speckle suppressor’, which contains a highly porous nanoberyllium plate squeezed between two beryllium windows. The insertion of the speckle suppressor in an X-ray beam allows manipulation of the spatial coherence length, thus changing the effective source size and removing the undesirable speckle structure in X-ray imaging experiments almost without beam attenuation. The absorption of the nanoberyllium plate is below 1% for 1 mm thickness at 12 keV. The speckle suppressor was tested on the ID06 ESRF beamline with X-rays in the energy range from 9 to 15 keV. It was applied for the transformation of the phase–amplitude contrast to the pure amplitude contrast in full-field microscopy.

## Abstract 2

A speckle suppression device containing highly porous nanoberyllium is proposed for manipulating the spatial coherence length and removing undesirable speckle structure during imaging experiments.

## 1. Introduction   

In recent years, synchrotron X-ray imaging and microscopy techniques have become a versatile and powerful tool in material science, biology, soft condensed matter physics and medicine. A high-brilliance, well collimated and coherent X-ray beam from a modern synchrotron source allows non-destructive investigation of low-contrast materials and interfaces with high resolution (Snigirev *et al.*, 1995 ▶; Cloetens *et al.*, 1996 ▶; Wilkins *et al.*, 1996 ▶; Gureyev *et al.*, 2009 ▶). At the same time, a complete characterization of the object is possible only by comparing the phase contrast image with the absorption image, obtained with fully incoherent irradiation. So, there is clearly a need for a device that could tailor the coherence properties of the X-ray source simply by insertion into an X-ray beam. Such a device should have a property allowing it to be quickly inserted and taken out from the X-ray beam without decreasing its flux intensity.

It is well known that a rotating random-phase-screen diffuser was employed on synchrotron X-ray imaging beamlines to ameliorate the X-ray field in time and space, as well as to blur out phase contrast artefacts introduced by the beamline optics and other components (Morgan *et al.*, 2010 ▶; Awaji, 2006 ▶). The use of a random phase screen also gives the possibility of tailoring the apparent source size, which is sometimes desired for imaging experiments. According to our knowledge, however, all known ultra dispersion systems are absorbing materials and the best transmission of 85% was achieved for amorphous boron powder (Matsuura *et al.*, 2004 ▶).

Here we present an X-ray diffuser based on highly porous nanoberyllium. The device, called a ‘speckle suppressor’, was manufactured and experimentally tested. The absorption of a 1 mm-thick nanoberyllium plate is below 1% at 12 keV X-ray energy. Inserting the speckle suppressor into the X-ray beam results in eliminating the phase artefacts caused by the beamline optics and other components in the final image of the sample; it also permits one to even out the X-ray field in time and space. The X-ray field thereby remains sensitive to sample-induced transverse phase gradients, whilst simultaneously being insensitive to transverse phase gradients which are not due to the sample.

As mentioned previously, the need for a diffuser stems from the difficulty in achieving a perfectly uniform X-ray beam for the full field of view using a synchrotron source. This is particularly evident when the area to be illuminated is relatively large (*e.g.* several millimetres to centimetres in extent), as required in biomedical small animal *in vivo* imaging (Matsuura *et al.*, 2004 ▶), where the frame-to-frame movement of the beam is particularly problematic. Generally, the use of such a speckle suppressor device would be helpful for any direct X-ray imaging technique, where a uniform X-ray beam is desired, while it would obviously be useless for any diffraction imaging approaches, like coherent diffraction imaging or ptychography.

## 2. Experimental results and discussion   

**Figure 1:**
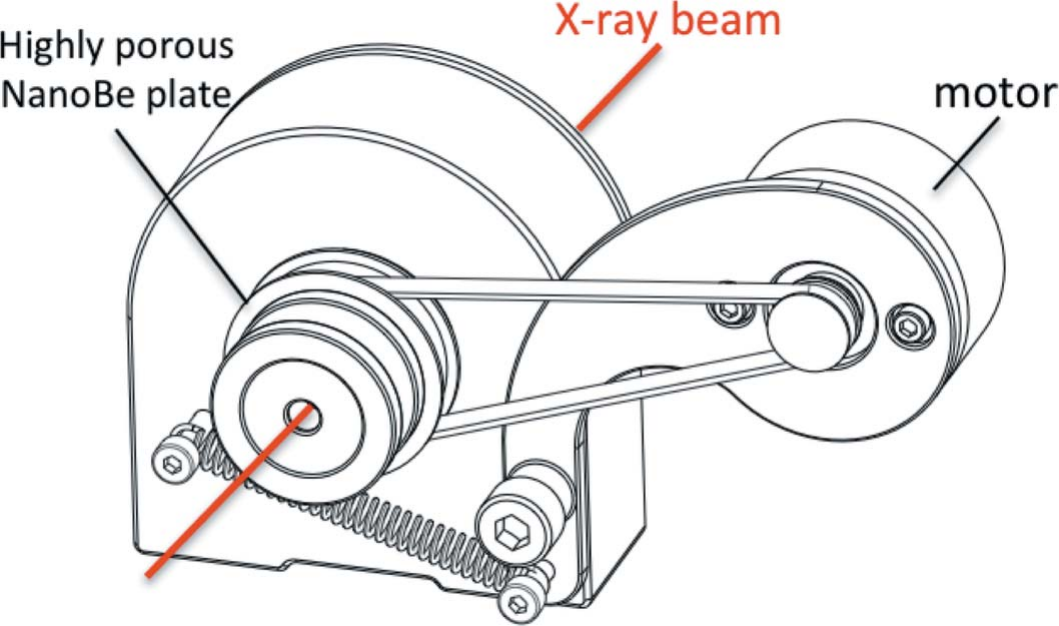
Sketch of the speckle suppressor device.

The highly porous nanoberyllium was made at Rosatom A. A. Bochvar High-Technology Scientific Research Institute for Inorganic Materials (Moscow, Russia) by hydration–dehydration technology (Gorlevsky *et al.*, 1995 ▶, 1998 ▶; Markushkin *et al.*, 2012 ▶). The pore dimensions vary from 0.1 µm up to 1 µm. The assembly of the diffuser, the speckle suppressor, is presented in Fig. 1 ▶. It contains a 4 mm-thick highly porous nanoberyllium plate, which was squeezed between two sapphire-coated beryllium windows. The plate was rotated at a constant speed of 200 r min^−1^ to create a uniform image. The rotation speed of 200 r min^−1^ was chosen assuming a full shift of the average speckle grain size (1 µm) at the time, which is certainly shorter than the minimum exposure time (0.05 s)

The speckle suppressor was tested at the Micro Optics Test Bench on the ID06 beamline of the ESRF. The beam was produced by an in-vacuum undulator and specified X-ray energies were selected by a cryogenically cooled Si(111) double-crystal monochromator. The vertical and horizontal source sizes were 40 and 900 µm (full width at half-maximum, FWHM), respectively. The speckle suppressor was tested for two practical applications: firstly, for manipulating the spatial coherence length; secondly, for the transformation of the phase–amplitude contrast image to the pure absorption one.

**Figure 2:**
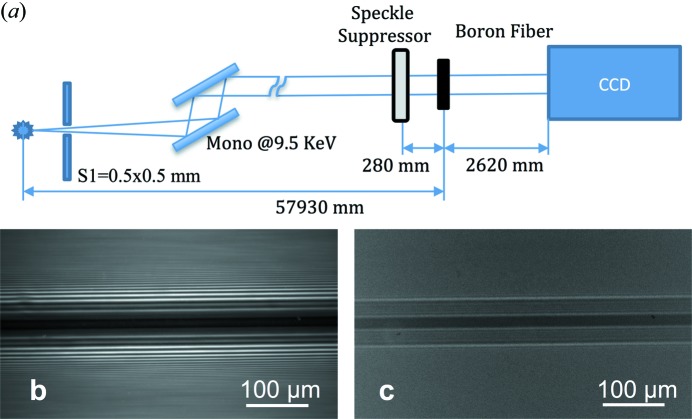
(*a*) Scheme of the boron fibre imaging experiment. (*b*) Phase contrast image of the boron fibre in the in-line imaging geometry. (*c*) Clear absorption contrast image of the boron fibre after insertion of the speckle suppressor.

In order to see the influence of the speckle suppressor on the effective source size or, in other words, the spatial coherence length, we performed an in-line phase contrast imaging experiment using boron fibre. The layout of the first experiment is depicted in Fig. 2 ▶(*a*). The monochromator was tuned to select X-ray energy 9.5 keV. The boron fibre was placed 57.93 m from the source. The speckle suppressor was located at 28 cm distance in front of the fibre. The images of the fibre with and without the speckle suppressor were detected with a high-resolution Sensicam CCD camera (0.6 µm pixel size), which was located at 2.6 m from the fibre.

The holographic image of the boron fibre without the speckle suppressor is shown in Fig. 2 ▶(*b*), where about 15 fringes are clearly seen. From the number of fringes and the distance between the last two resolvable fringes we can estimate the effective source size, which is in the order of 50 µm, with the spatial coherence length of 100 µm (see Kohn, http://X-ray-optics.ucoz.ru/editor.htm). When the speckle suppressor was introduced in the beam, the interference fringes disappeared, leaving only the absorption contrast image with the first-order fringes (Fig. 2 ▶ *c*). With the insertion of the speckle suppressor, the effective source size, calculated according to the program available at http://X-ray-optics.ucoz.ru/js-pro/list.htm, increased fourfold and thus became 200 µm.

**Figure 3:**
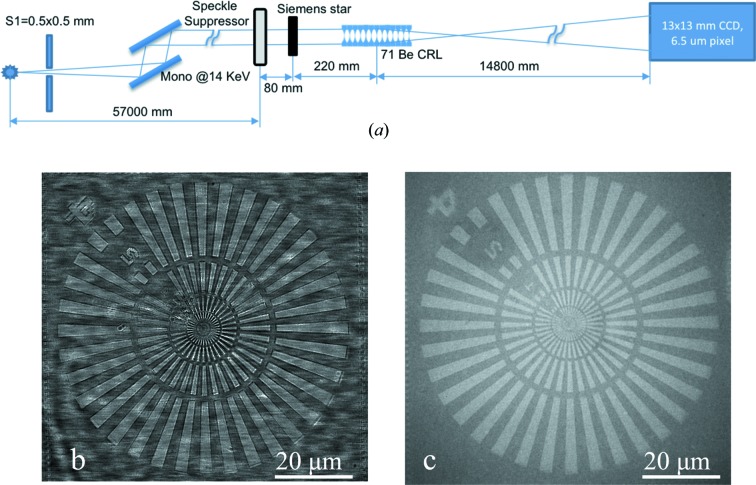
(*a*) Scheme of microscopy setup with 71 Be lenses; (*b*) magnified image of the Siemens star object without the speckle suppressor; (*c*) magnified image after introducing the speckle suppressor 10 mm before the Siemens star.

To demonstrate the ability of the speckle suppressor to manipulate the contrast transformation we used a full-field X-ray microscopy setup at 14 keV (Fig. 3 ▶ *a*). An objective lens assembly of 71 individual Be parabolic lenses with a 50 µm radius of parabola apex was located 57.3 m from the source. A Siemens star test object was placed on the translation/rotation stage with the translation direction along the beam axis, which was located 220 mm in front of the lens. The Photonic Science CCD detector with the field of view in the order of 13 mm and pixel size of 6.5 µm was placed 14.8 m downstream of the objective lens. Thus, the magnification factor of 67 was achieved. The speckle suppressor was placed 80 mm upstream of the test object.

The X-ray images of the Siemens star without and with the speckle suppressor are presented in Figs. 3 ▶(*b*) and 3 ▶(*c*), respectively. The image formation without the speckle suppressor relies on the mixture of the amplitude and phase contrast, which is why a lot of artefacts and speckles coming from the Be lenses are seen. It should be noted that the type of Be used for the lenses (O30H) in the experiment is the purest material commercially available. By introducing the speckle suppressor in the beam, the image is formed only by absorption, and is very clear.

The calculation of the average integral intensity ratio in both images showed a 34% loss of the total intensity by using the speckle suppressor, caused mostly by small-angle X-ray scattering coming from porous nanoberyllium. Taking into account the pore sizes, it can be easily estimated that scattering angles are within the 0.1–1 mrad range (Guinier, 1963 ▶). To measure the real absorption of the porous nanoberyllium plate, the flat-field intensity measurements with and without the speckle suppressor were performed with the CCD detector. The results have shown that the total absorption by the speckle suppressor device (two 200 µm-thick Be windows and a 4 mm porous nanoberyllium plate) does not exceed 3%. From this it follows that ∼31% of the intensity loss in the microscopy is accounted for by diffuse or small-angle scattering.

Thus, the optimal position of the device should be chosen considering the angular scattering issue for the porous nanoberyllium, depending on the pore sizes. Therefore it does not make sense to move the device very far from the sample in the microscopy scheme. The optimal use of the speckle suppressor is in X-ray projection microscopy, where the lens angular acceptance is wider than the difuse scattering angle from porous nanoberyllium. This scheme was realized with the speckle suppressor located at the focus position of the same stack of 71 Be (O30H) lenses at 14.4 keV. The ratio of the average integral intensity with and without the speckle suppressor was calculated as 77%, meaning that in such an arrangement the intensity loss due to the scattering was only 20%.

## 3. Computation theory associated with porous nanoberyllium   

Correct simulation of the intensity of X-rays passing through porous nanoberyllium is complicated due to the difficulty associated with specifying the appropriate parameters. An example of a software program which allows the insertion of defects in the lens is *SRW* (Chubar *et al.*, 2002 ▶).^1^ With the help of this program it is possible to simulate defects of spherical symmetry. Porous nanoberyllium has a density of 0.2 g cm^−3^; however, the density of ordinary beryllium is almost tenfold that, 1.85 g cm^−3^. This means that, in order to properly simulate a porous nanoberyllium plate with the dimensions 1 × 1 × 1 mm, the plate should be formed with almost 90% of empty spaces. To simulate a plate with 0.1–1 µm-diameter empty spaces using *SRW* for such a beryllium lens, from 9 × 10^8^ to 9 × 10^11^ defects must be inserted. Such a task would be impossible even with the help of supercomputers. Because of the amount of calculations involved, and in order to enable the simulation of a porous nanoberyllium plate, its model, while retaining all the required properties, must be simplified.

The most efficient way of solving this problem is to create an appropriate statistical model. Approximately 90% of the plate should consist of empty space, so in the model only 10% of the plate should be made of beryllium. Such a sample can be simulated by dividing the portion of the considered material into smaller pieces and making a random choice, so that there are both empty spaces and matter. Obviously, the size of these parts should be properly linked with the size of the pore. For porous nanoberyllium, whose cavities have a diameter of 0.1–1 µm, the statistical model should consist of same-scale elements. This is reasonable, since, statistically, if we consider smaller-sized elements, those which are located close to each other will have a greater impact on the changes in the X-ray intensity than would be indicated by the size of the element. On the other hand, the choice of elements with a diameter larger than 1 µm will cause insignificant changes in the probability of occurrence of empty space or material, respectively.

Another important issue that must be taken into account in the calculations is the selection of an appropriate model, which would enable the calculation of the X-ray intensity inside the porous beryllium material, and in the air after X-rays pass through the material. The classical methods based on the use of the Fourier transform technique for the analytical solution of the paraxial wave equation (Duffieux, 1983 ▶; Goodman, 2005 ▶; Wilson, 1995 ▶; Scott, 1998 ▶) do not allow one to carry out this kind of calculation due to the discontinuity of the data. For this reason, the simulation scheme was based on the finite difference method, which does not require continuity or differentiability from the data. Propagation of the X-ray waves through optical objects is described by
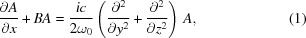
where *B* is the function which corresponds to the form of the beryllium plate. The developed method is built by means of discretization of equations of monochromatic electromagnetic wave propagation (1) with respect to spatial variables, which are perpendicular to the optical axis of the optic system. Approximate ordinary differential equations resulting from the discretization of the system of ordinary differential equations are solved with the help of the implicit Runge–Kutta method of the second order, combined with an iterative procedure.

The simulation was carried out for a plate of size 0.3 × 0.3 × 5 mm; the surface perpendicular to the propagation of X-rays has the size of 0.3 × 0.3 mm. In order to obtain a plate model, the entire plate is divided into 900 × 900 × 15000 pieces (cubes). To obtain a model of the beryllium plate without pores we suggest that all the cubes are composed entirely of beryllium. The density of the porous beryllium plate must be about ten times smaller, so only 1/10 of the elements should have the properties of beryllium. Therefore, for each cube we must select whether the element corresponds to either a beryllium-like element (with probability 1/10), or an empty space (with probability 9/10). To obtain the distribution of beryllium in the plate for a one-step numerical procedure along the *x* axis, such a selection should be repeated independently 300 times. Thus, in accordance with the central limit theorem, for each of the 900 × 900 points in the plane (*y*, *z*) we obtain the simulation of a plate with a thickness of 0.1 mm, whose density is ten times lower than that of the plate made entirely of beryllium, and wherein the average location of elements (voids and spaces containing beryllium) will be distributed according to the normal distribution. If the average density distribution of beryllium in the plate is known, it is possible to determine the values of the function *B* for each spatial step of the numerical procedure. In order to obtain results for the entire plate, 50 steps of the numerical procedure need to be performed. Of course, the selection of elements for each of these steps must be performed independently. The postulated model has been proposed for a plate with a 1 µm pore size, and should be properly adjusted when plates with smaller pores are considered.

**Figure 4:**
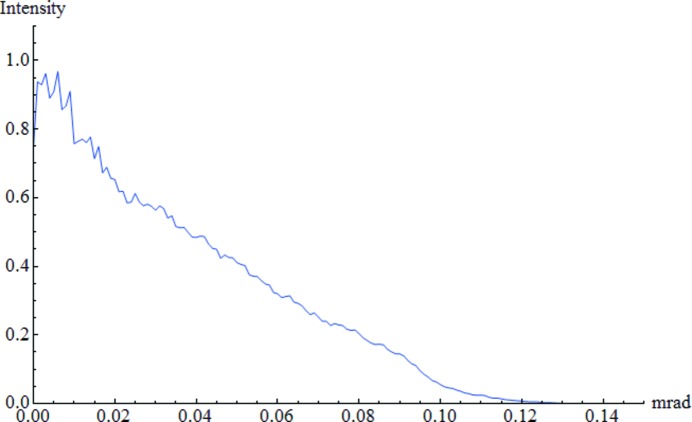
The scattering of X-ray waves of initial energy 12.4 keV obtained for cavities simulated by elements, which have a size of 1/3 × 1/3 × 1/3 µm.

Fig. 4 ▶ presents the scattering of X-ray waves of initial energy 12.4 keV obtained for cavities simulated by elements with a size of 1/3 × 1/3 × 1/3 µm. Such a model of porous nanoberyllium gives X-ray scattering of the order of 0.1 mrad. Inclusion of smaller components increases the average angular scattering, and larger ones cause a reduction of scattering. Accordingly, the X-ray small-angle scattering increases linear­ly for the simulations carried out for smaller elements. It means that in order to obtain scattering of the order of 1 mrad, the porous nanoberyllium plate should be simulated with the help of elements whose approximate size is 1/30 × 1/30 × 1/30 µm. The obtained result is expected because it shows that for the simulation of porous material the number of elements needed to simulate a single defect should be at least equal to 27 (3 × 3 × 3); otherwise, it will be impossible for the model to faithfully resemble the structure of porous nanoberyllium. Due to large scattering it is not possible to carry out calculations at large distances from the beryllium plate, because this involves increasing the area of computation that must be taken into account, and inevitably lengthens the computation time. Along with the increase of the distance from the beryllium plate, the number of steps in the finite difference method also grows. This, in turn, reduces the accuracy of the results, and is the reason why this method is only suitable for achieving statistical results. The possibility of accurate X-ray imaging for porous plates of nanoberyllium is a serious mathematical problem in need of further investigation.

## 4. Conclusions   

A speckle suppressor device based on highly porous nanoberyllium was applied for manipulating the spatial coherence length by increasing the effective source size and by the transformation of the contrast mechanism during the imaging experiments. From the experiments performed the optimal position of the speckle suppressor was defined: for X-ray projection microscopy the device has to be placed exactly at the secondary source position; for full-field microscopy with lenses it has to be located in the lens imaginary focus; and for radiography schemes the speckle suppressor has to be positioned just in front of the object. However, the X-ray small-angle scattering in the ∼0.1–1 mrad angular range, due to the pores, has to be taken into account, considering the use of speckle suppression. Higher-energy applications of the speckle suppressor require an increase in the nanoberyllium material density and a reduction of the average porous size.

## Footnotes

1
*Synchrotron Radiation Workshop* (*SRW*) is electrodynamics/physical optics computer code for calculation of synchrotron radiation and simulation of fully and partially coherent radiation wavefront propagation.

## References

[bb1] Awaji, M. (2006). *IPAP Conf. Ser.* **7**, 157–158.

[bb2] Chubar, O., Elleaume, E., Kuznetsov, S. & Snigirev, A. (2002). *Proc. SPIE*, **4769**, 145.

[bb3] Cloetens, P., Barrett, R., Baruchel, J., Guigay, J. P. & Schlenker, M. (1996). *J. Phys. D*, **29**, 133–146.

[bb4] Duffieux, P. M. (1983). *The Fourier Transform and its Applications to Optics.* New York: Wiley.

[bb5] Goodman, J. (2005). *Introduction to Fourier Optics*, 3rd ed. Greenwood Village: Roberts and Co.

[bb6] Gorlevsky, V. V., Kostylev, F. A., Starshina, V. G., Senin, M. D., Golikov, I. V., Kondratyev, M. V. & Chubakova, T. A. (1995). *Izv. Acad. Nauk. Ser. Neorg. Mater.* **31**, 479–482.

[bb7] Gorlevsky, V. V., Markushkin, Yu. E. & Petrunin, V. F. (1998). *J. Moscow Phys. Soc.* **8**, 373–376.

[bb8] Guinier, A. (1963). *X-ray Diffraction.* San Francisco: W. H. Freeman.

[bb9] Gureyev, T. E., Mayo, S. C., Myers, D. E., Nesterets, Y., Paganin, D. M., Pogany, A., Stevenson, A. W. & Wilkins, S. W. (2009). *J. Appl. Phys.* **105**, 102005–102012.

[bb10] Markushkin, Yu. E., Gorlevsky, V. V., Zabrodin, A. V., Tuzov, Yu. V., Morozov, I. A., Brylev, D. A., Lizunov, A. V., Shevyrdyaev, M. S., Satanovskiy, A. V., Demin, A. V., Semenov, A. A., Aleksandrov, P. A. & Belova, N. E. (2012). *VANT, seriya: Materialovedenie i nov. Material*, **72**, 130–136.

[bb11] Matsuura, Y., Yoshizaki, I. & Tanaka, M. (2004). *J. Appl. Cryst.* **37**, 841–842.

[bb12] Morgan, K. S., Irvine, S. C., Suzuki, Y., Uesugi, K., Takeuchi, A., Paganin, D. M. & Siu, K. K. W. (2010). *Opt. Commun.* **283**, 216–225. 10.1364/OE.18.013478 20588478

[bb13] Scott, C. (1998). *Introduction to Optics and Optical Imaging.* New York: Wiley.

[bb14] Snigirev, A., Snigireva, I., Kohn, V., Kuznetsov, S. & Schelokov, I. (1995). *Rev. Sci. Instrum.* **66**, 5486–5492.

[bb15] Wilkins, S. W., Gureyev, T. E., Gao, D., Pogany, A. & Stevenson, A. W. (1996). *Nature (London)*, **384**, 335–338.

[bb16] Wilson, R. (1995). *Fourier Series and Optical Transform Techniques in Contemporary Optics.* New York: Wiley.

